# RTA 408 Inhibits Interleukin-1β-Induced MMP-9 Expression via Suppressing Protein Kinase-Dependent NF-κB and AP-1 Activation in Rat Brain Astrocytes

**DOI:** 10.3390/ijms20112826

**Published:** 2019-06-10

**Authors:** Chien-Chung Yang, Chih-Chung Lin, Mei-Jie Jou, Li-Der Hsiao, Chuen-Mao Yang

**Affiliations:** 1 Department of Traditional Chinese Medicine, Chang Gung Memorial Hospital at Tao-Yuan, Kwei-San, Tao-Yuan 333, Taiwan; r55161@cgmh.org.tw; 2 School of Traditional Chinese Medicine, College of Medicine, Chang Gung University, Kwei-San, Tao-Yuan 333, Taiwan; 3 Department of Anesthetics, Chang Gung Memorial Hospital at Linkuo, and College of Medicine, Chang Gung University, Kwei-San, Tao-Yuan 333, Taiwan; chihchung@adm.cgmh.org.tw (C.-C.L.); lidesiao@livemail.tw (L.-D.H.); 4 Department of Physiology and Pharmacology and Health Ageing Research Center, College of Medicine, Chang Gung University, Kwei-San, Tao-Yuan 333, Taiwan; mjjou@mail.cgu.edu.tw; 5 Research Center for Industry of Human Ecology and Graduate Institute of Health Industry Technology, Chang Gung University of Science and Technology, Tao-Yuan 333, Taiwan

**Keywords:** neuroinflammation, astrocytes, IL-1β, matrix metalloproteinase, Chinese herbal medicine

## Abstract

Neuroinflammation is characterized by the elevated expression of various inflammatory proteins, including matrix metalloproteinases (MMPs), induced by various pro-inflammatory mediators, which play a critical role in neurodegenerative disorders. Interleukin-1β (IL-1β) has been shown to induce the upregulation of MMP-9 through nicotinamide adenine dinucleotide phosphate (NADPH) oxidase (NOX)-reactive oxygen species (ROS)-dependent signaling pathways. N-(2-cyano-3,12-dioxo-28-noroleana-1,9(11)-dien-17-yl)-2-2-difluoropropanamide (RTA 408), a novel synthetic triterpenoid, has been shown to possess anti-oxidant and anti-inflammatory properties in various types of cells. Here, we evaluated the effects of RTA 408 on IL-1β-induced inflammatory responses by suppressing MMP-9 expression in a rat brain astrocyte (RBA-1) line. IL-1β-induced MMP-9 protein and mRNA expression, and promoter activity were attenuated by RTA 408. The increased level of ROS generation in RBA-1 cells exposed to IL-1β was attenuated by RTA 408, as determined by using 2′,7′-dichlorodihydrofluorescein diacetate (DCFH-DA) and CellROX. In addition, the inhibitory effects of RTA 408 on MMP-9 expression resulted from the suppression of the IL-1β-stimulated activation of Pyk2 (proline-rich tyrosine kinase), platelet-derived growth factor receptor β (PDGFRβ), Akt, ROS, and mitogen-activated protein kinases (MAPKs). Pretreatment with RTA 408 attenuated the IL-1β-induced c-Jun phosphorylation, mRNA expression, and promoter activity. IL-1β-stimulated nuclear factor-κB (NF-κB) p65 phosphorylation, translocation, and promoter activity were also attenuated by RTA 408. Furthermore, IL-1β-induced glial fibrillary acidic protein (GFAP) protein and mRNA expression, and cell migration were attenuated by pretreatment with RTA 408. These results provide new insights into the mechanisms by which RTA 408 attenuates IL-1β-mediated inflammatory responses and exerts beneficial effects for the management of brain diseases.

## 1. Introduction

Neuroinflammatory processes are involved in the pathogenesis of neurodegenerative diseases, such as Parkinson’s disease (PD), Alzheimer’s disease (AD), Huntington’s disease, amyotrophic lateral sclerosis, and progressive supranuclear palsy [1]. In several animal models of neurodegenerative diseases and in human studies, cytokines and chemokines have been verified as risk factors [2,3]. Among these cytokine responses, the pro-inflammatory cytokine interleukin-1 (IL-1) plays a key role in the pathology of neurodegeneration [4]. In particular, IL-1β has important homeostatic functions in healthy organisms, such as in the regulation of feeding, sleep, and temperature [5]. However, the overproduction of IL-1β has been implicated in the pathophysiological changes that occur during different disease states, such as rheumatoid arthritis, vascular disease, multiple sclerosis, AD, and PD [3]. In the central nervous system (CNS), IL-1β can be released from various types of cells, including Schwann cells, microglia, and astrocytes [6,7]. Upon binding to IL-1 receptors, IL-1β activates the sequential phosphorylation of protein kinases, such as platelet-derived growth factor receptor (PDGFR), Pyk2, c-Src, phosphoinositide 3-kinase (PI3K)/Akt, and mitogen-activated protein kinases (MAPKs), and nuclear factor κB (NF-κB) signaling, which leads to the expression of various target genes by transcriptional and posttranscriptional mechanisms [8,9]. Astrocytes are specialized glial cells that play many important roles in the CNS, such as nourishing neurons, regulating the homeostasis of the extracellular space, regulating the metabolism of neurons, modulating synaptic signaling, and integrating the blood-brain barrier (BBB). Astrocytes can also promote the survival of neurons and other glia by releasing neurotrophic and gliotrophic factors. However, in contrast, they can release inflammatory mediators, such as tumor necrosis factor (TNF)-α, IL-1β, and matrix metalloproteinases (MMPs), involved in the pathological processes of brain insults [6,10]. The upregulation of MMP-9, in particular, participates in the pathogenesis of BBB breakdown, demyelination, inflammation, and neurotoxicity [11,12,13]. Furthermore, IL-1β-mediated inflammatory responses have been shown to induce the expression and activation of MMP-9 via c-Src, PDGFR, PI3K/Akt, Nox2/ROS, and the MAPKs-dependent activation of NF-κB and activator protein 1 (AP-1) [9,14], thus contributing to neuronal degeneration [15]. Reactive oxygen species (ROS) generation is also involved in MMP-9 induction in various types of cells [9,16]. Therefore, the development of anti-oxidant and anti-inflammatory drugs targeting these intracellular signaling pathways to attenuate MMP-9 expression may be beneficial for the management of neurodegenerative disorders.

Reactive gliosis is characterized by the formation of glial cell soma and the accumulation and activation of enlarged glial cells, notably astrocytes and microglia [17,18]. In the pathological processes of brain inflammation, activated glia harm neuron through creating neurotoxic factors such as glutamate, S100B, TNFα, prostaglandins, ROS, and reactive nitrogen species [19]. These inflammatory mediators provoke adjacent cells, including astrocytes and endothelial cells, amplifying inflammation through a vicious cycle of the autocrine and paracrine and leading to neuronal damage and diseases. Although the exact mechanisms of these inflammatory responses induced by reactive gliosis in disease progression are still poorly understood, reactive gliosis may also be a potential target to prevent the progression of neurodegenerative disorders.

Herbal medicines are well known as complementary and alternative agents that provide new therapeutic strategies for the management of neurodegenerative diseases. Among herbal medicines, oleanolic acid is a naturally occurring pentacyclic triterpenoid related to betulinic acid [20]. During the last decade, different triterpenoid compounds that display beneficial therapeutic effects on various disease conditions have been isolated from various plants and herbs [21]. In particular, RTA 408, a synthetic triterpenoid compound, binds to Keap1, which attenuates the degradation of Nrf2 and suppresses the generation of NO and pro-inflammatory cytokines in macrophages stimulated by IFN-γ [22]. In addition, RTA 408 exerts anti-inflammatory effects by inhibiting the pro-inflammatory transcription factor NF-κB and increasing the expression of Nrf2-targeted genes, including NQO1, TXNRD1, and GCLC [22,23,24,25]. The anti-inflammatory activity of RTA 408 has also been demonstrated in models of radiation-induced dermatitis [24,25,26], chronic diabetic wounds [27], renal ischemia-reperfusion injury [28], epilepsy [29], and in tumor cell lines [22]. However, whether RTA 408 exerts anti-inflammatory effects via suppressing IL-1β-induced MMP-9 expression in neuroinflammatory disorders is still unknown. Therefore, experiments were conducted to investigate the effects of RTA 408 on cell migration, astrogliosis, and the signaling pathways involved in MMP-9 expression induced by IL-1β in RBA-1 cells. Our results demonstrate that RTA 408 attenuates IL-1β-induced MMP-9 expression and cell migration via the suppression of Pyk2, PDGFRβ, PI3K/Akt, ROS, MAPKs, c-Jun, and NF-κB signaling components in RBA-1 cells. The anti-inflammatory effects of RTA 408 may provide an effective intervention to protect against neurodegeneration and neuroinflammation in brain diseases.

## 2. Results

### 2.1. RTA 408 Attenuates IL-1β-Induced MMP-9 Expression

**Figure 1:**
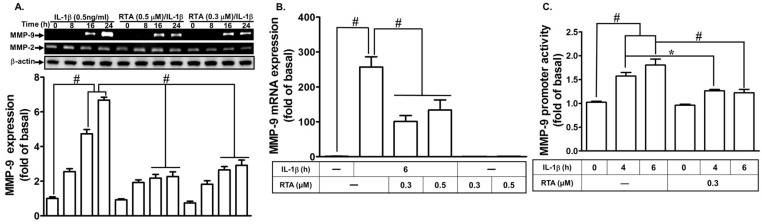
RTA 408 attenuates proMMP-9 expression induced by IL-1β. (**A**) Cells were pretreated with RTA 408 (0.3, and 0.5 µM) for 1 h, and then exposed to IL-1β (0.5 ng/mL) for the indicated time intervals (8, 16, and 24 h). The MMP-9 level was determined by gelatin zymography. The β-actin level of cell lysates was assayed by western blot. (**B**) Cells were incubated with RTA 408 (0.3, and 0.5 µM) for 1 h, and then incubated with IL-1β (0.5 ng/mL) for 6 h. The mRNA levels of MMP-9 were determined by real-time RT-PCR. (**C**) Cells were incubated with RTA 408 (0.3 µM) for 1 h, and then incubated with IL-1β (0.5 ng/mL) for the indicated time intervals (4 and 6 h). The promoter activity of MMP-9 was determined by promoter reporter assay kit. Data are expressed as the mean ± standard error of the mean (SEM; *n* = 3). * *p* < 0.05; ^#^ *p* < 0.01, as compared with the cells stimulated with IL-1β only or compared between the indicated groups. The figure represents one of three individual experiments.

We explored whether RTA 408 inhibits IL-1β-induced MMP-9 expression. As shown in Figure 1A, IL-1β-induced MMP-9 activity was attenuated by pretreatment with RTA 408 within 16–24 h, as determined by gelatin zymography. To determine whether the level of transcriptional activity was involved in the inhibitory effects of RTA 408 on IL-1β-induced MMP-9 expression, the levels of MMP-9 mRNA expression and promoter activity were measured by real-time PCR and a promoter activity assay, respectively. We found that pretreatment with RTA 408 attenuated IL-1β-induced MMP-9 mRNA expression (Figure 1B) and promoter activity (Figure 1C). These results suggest that RTA 408 indeed blocks IL-1β-induced MMP-9 expression at the transcriptional level in RBA-1 cells.

### 2.2. RTA 408 Inhibits IL-1β-Stimulated Phosphorylation of Pyk2/PDGFR/Akt in RBA-1 Cells

**Figure 2:**
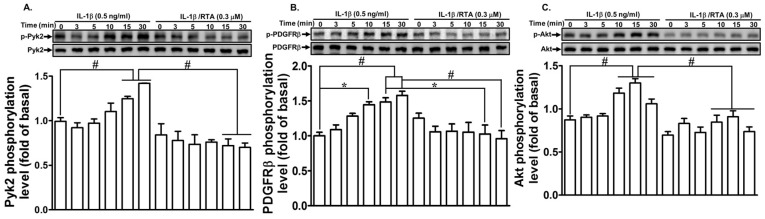
RTA 408 attenuates the IL-1β-induced proMMP-9 expression via suppressing the phosphorylation of Pyk2, platelet-derived growth factor receptor β (PDGFRβ), and Akt. Cells were pretreated with RTA 408 (0.3 µM) for 4 h, and then incubated with IL-1β (0.5 ng/mL) for the indicated time intervals (3, 5, 10, 15, and 30 min). The cell lysates were assayed by western blot to detect the phosphorylation of Pyk2 (**A**), PDGFRβ (**B**), and Akt (**C**) using their respective phosphorylated antibody. Data analysis and processing are described in the section “Statistical Analysis of Data.” * indicates *p* < 0.05; # indicates *p* < 0.01, as compared between the indicated groups. The figure represents one of three individual experiments.

Nonreceptor tyrosine kinases such as Pyk2 are involved in several cellular functions evoked by various stimuli [30,31]. They are involved in regulatory mechanisms critical to various physiological processes, including cell growth, differentiation, metabolism, cell cycle regulation, and cytoskeleton function. Here, we investigated whether RTA 408 blocks Pyk2 phosphorylation leading to reduction in MMP-9 expression induced by IL-1β. RBA-1 cells were pre-incubated with RTA 408 for 1 h and then exposed to IL-1β for the indicated time intervals. As shown in Figure 2A, IL-1β time-dependently stimulated the phosphorylation of Pyk2, with a maximal response within 10–30 min, and this effect was attenuated by pretreatment with RTA 408.

In addition, receptor tyrosine kinases such as epidermal growth factor receptor (EGFR) and platelet-derived growth factor receptor (PDGFR) are activated either by interactions with their ligands or through a ligand-independent transactivation process [32]. PDGFs and their receptors have been intensely investigated and play pivotal roles in normal development and pathologies of human diseases [33]. Our previous study revealed that PDGFR is involved in IL-1β-mediated responses [9]. Thus, we clarified whether RTA 408 attenuates IL-1β-induced MMP-9 expression via blocking PDGFR phosphorylation. We found that IL-1β stimulated the phosphorylation of PDGFRβ in a time-dependent manner and reached a maximal response within 10–30 min. This effect was attenuated by pretreatment with RTA 408 (Figure 2B).

Akt is a common downstream target of PDGFR and plays an important role in various cellular functions, including metabolism, proliferation, survival, growth, angiogenesis, migration, and invasion [34]. Akt has been shown to be involved in IL-1β-mediated responses [35]. Thus, we investigated whether RTA 408 interferes with Akt and blocks IL-1β-mediated responses. As shown in Figure 2C, IL-1β stimulated the phosphorylation of Akt in a time-dependent manner and reached a maximal response within 10–30 min. This effect was attenuated by pretreatment with RTA 408. These results suggest that RTA 408 attenuates IL-1β-induced MMP-9 expression via suppressing the phosphorylation of Pyk2/PDGFR/Akt signaling in RBA-1 cells.

### 2.3. RTA 408 Inhibits IL-1β-Stimulated ROS Generation in RBA-1 Cells

**Figure 3:**
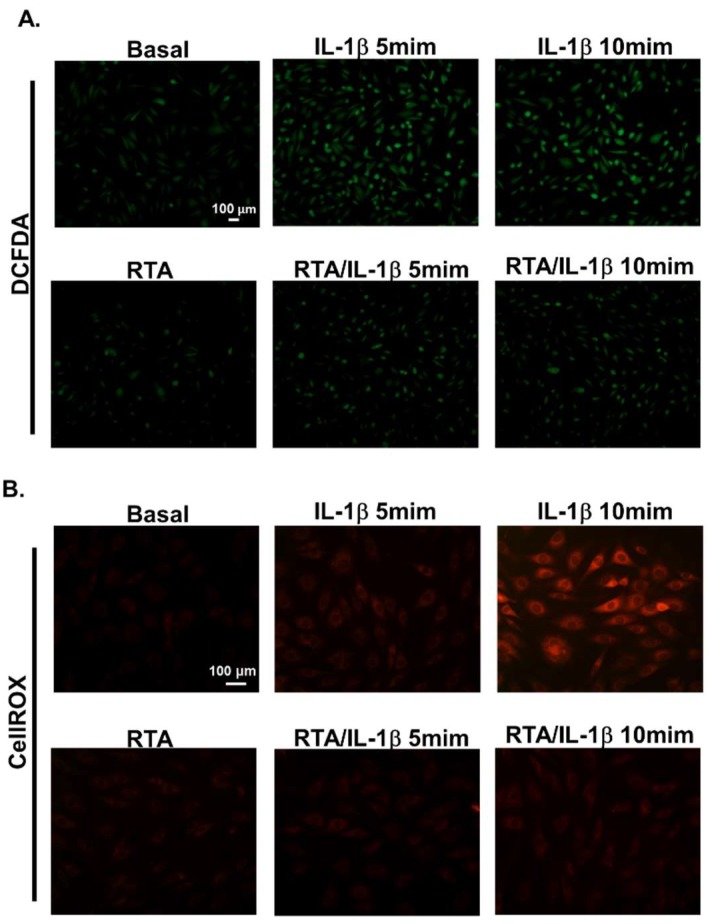
Reactive oxygen species (ROS) involved in the inhibited mechanisms of RTA 408 on the IL-1β response in RBA-1 cells. Cells on the coverslips were pretreated with/without RTA 408 (0.3 µM) for 4 h, and then incubated with IL-1β (0.5 ng/mL) for the indicated time intervals (5 and 10 min). Then, cells were incubated with 10 µM DCFH-DA (**A**) and 5 µM CellROX Deep Red Reagent (**B**) for 30 min, respectively, the fluorescence intensity was detected under a fluorescence microscope. The figure represents one of three individual experiments.

MMPs expression could be regulated by ROS in various cell types [16,36]. The findings of our previous studies also confirmed that IL-1β-induced MMP-9 expression and cell migration are mediated via NADPH oxidase 2-derived ROS signals [9]. Here, we investigated whether RTA 408 attenuates ROS generation and thus blocks IL-1β-mediated MMP-9 expression. RBA-1 cells were pretreated with RTA 408 (0.3 µM) for 4 h and then exposed to IL-1β (0.5 ng/mL) for the indicated time intervals (5 and 10 min). We found that IL-1β-stimulated ROS generation was attenuated by pretreatment with RTA 408 (Figure 3A,B). These results confirmed that RTA 408 inhibits IL-1β-induced MMP-9 expression via the attenuation of ROS generation in RBA-1 cells.

### 2.4. RTA 408 Inhibits IL-1β -Stimulated Phosphorylation of MAPKs in RBA-1 Cells

**Figure 4:**
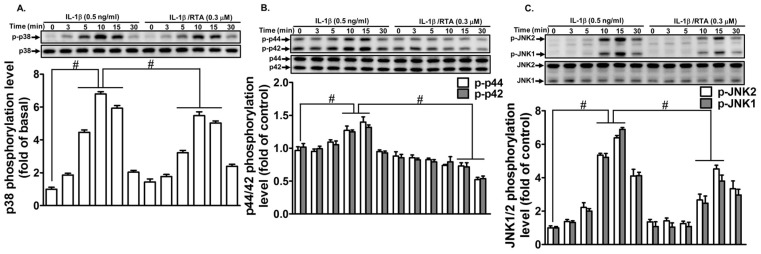
RTA 408 attenuates the IL-1β-induced proMMP-9 expression via suppressing MAPKs phosphorylation. Cells were pretreated with RTA 408 (0.3 µM) for 4 h, and then incubated with IL-1β (0.5 ng/mL) for the indicated time intervals (3, 5, 10, 15, and 30 min). The cell lysates were assayed by western blot to detect phosphorylation of p38 MAPK (**A**), p42/p44 MAPK (**B**), and JNK1/2 (**C**) using their respective phosphorylated antibody. Data analysis and processing are described in the section “Statistical Analysis of Data.” # indicates *p* < 0.01, as compared between the indicated groups. The figure represents one of three individual experiments.

MAPKs, including extracellular signal-regulated kinase 1/2 (ERK1/2), c-Jun amino-terminal kinase 1/2 (JNK1/2), and p38 MAPK, have been shown to regulate various pathological processes of human diseases and cellular functions [37]. Our previous studies have indicated that MAPKs regulate the IL-1β-induced MMP-9 expression [38]. Thus, we explored whether RTA 408 interferes with MAPKs and blocks IL-1β-mediated MMP-9 expression. As shown in Figure 4A–C, one hour after RTA 408 (0.3 µM) treatment, RBA-1 cells were exposed to IL-1β (0.5 ng/mL) for the indicated time intervals. The levels of p38 MAPK (Figure 4A), p42/p44 MAPK (Figure 4B), and JNK1/2 phosphorylation (Figure 4C) were decreased compared with those after IL-1β stimulation alone. These results suggest that RTA 408 attenuates IL-1β-induced MMP-9 expression via the suppression of MAPK phosphorylation in RBA-1 cells.

### 2.5. RTA 408 Inhibits IL-1β-Stimulated Activation of NF-κB and AP-1 in RBA-1 cells

**Figure 5:**
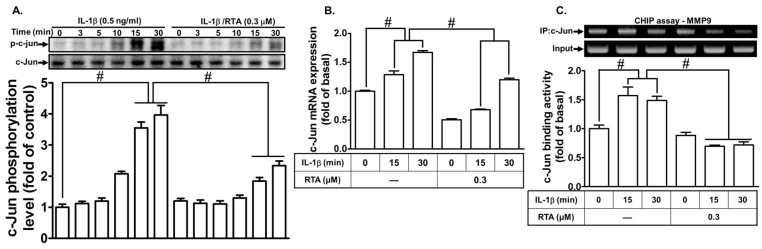
RTA 408 attenuates the IL-1β-induced proMMP-9 expression via down-regulating AP-1 activities. (**A**) Cells were pretreated with RTA 408 (0.3 µM) for 4 h, and then incubated with IL-1β (0.5 ng/mL) for the indicated time intervals (3, 5, 10, 15, and 30 min), the phosphorylation of c-Jun was detected by western blot. (**B**) Cells were pretreated with RTA 408 (0.3 µM) for 4 h, and then incubated with IL-1β (0.5 ng/mL) for the indicated time intervals (15 and 30 min). The mRNA levels of c-Jun were determined by real-time RT-PCR. (**C**) Cells were pretreated with/without RTA 408 (0.3 µM) for 4 h, and then incubated with IL-1β (0.5 ng/mL) for the indicated time intervals (15 and 30 min), ChIP assay was performed by using a specific antibody of c-Jun. Data analysis and processing are described in the section “Statistical Analysis of Data.” # indicates *p* < 0.01, as compared between the indicated groups. The figure represents one of three individual experiments.

Several lines of evidence have shown that the transcription factors NK-κB and AP-1 have DNA-binding domains that bind with specific sequences in MMP-9 promoters, leading to MMP-9 gene transcription [39]. Several previous reports have shown that NK-κB and AP-1 are involved in IL-1β-induced MMP-9 expression in different cells, including RBA-1 cells [9,40,41]. Therefore, we explored whether RTA 408 attenuates IL-1β-induced MMP-9 expression via suppressing NK-κB and AP-1 activation. As shown in Figure 5A, the IL-1β-stimulated phosphorylation of c-Jun was inhibited by pretreatment with RTA 408. We further determined whether RTA 408 attenuates IL-1β-induced MMP-9 expression through the downregulation of AP-1. As shown in Figure 5B, we found that RTA 408 attenuated c-Jun mRNA expression induced by IL-1β in RBA-1 cells. To confirm that the interaction between c-Jun and the MMP-9 promoter is blocked by RTA 408, a ChIP assay was performed. As shown in Figure 5C, the cells were pretreated with RTA 408 for 1 h and then stimulated by IL-1β for the indicated time intervals (15 and 30 min). The results indicated that the interaction between c-Jun and the MMP-9 promoter was blocked by pretreatment with RTA 408.

**Figure 6:**
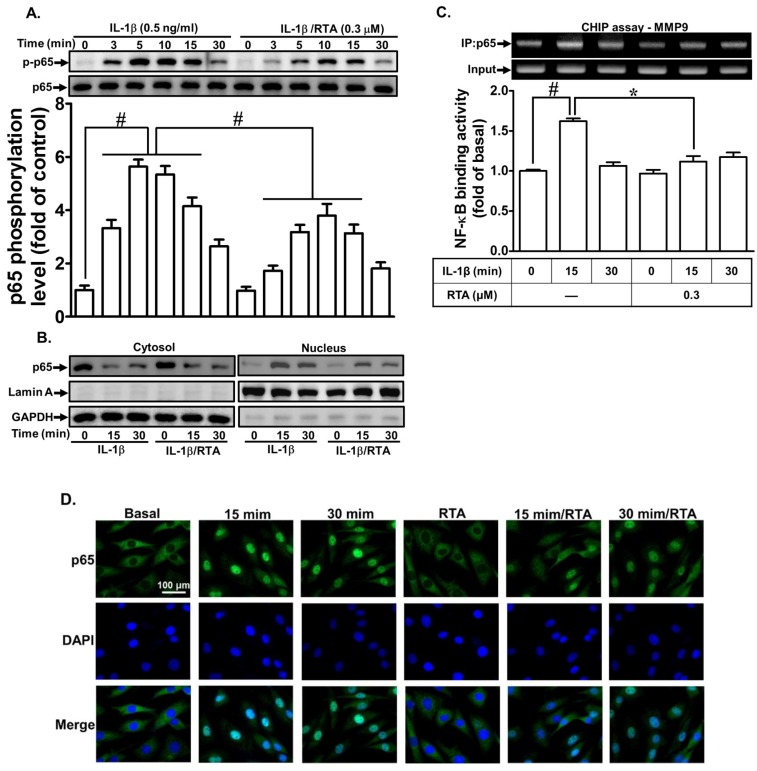
RTA 408 attenuates the IL-1β-induced proMMP-9 expression via down-regulating NF-κB activities. (**A**) Cells were pretreated with RTA 408 (0.3 µM) for 4 h, and then incubated with IL-1β (0.5 ng/mL) for the indicated time intervals (3, 5, 10, 15, and 30 min), the phosphorylation of p65 was detected by western blot. (**B**) Cells were pretreated with or without RTA 408 (0.3 µM) for 4 h and incubated with 0.5 ng/mL IL-1β for the indicated time intervals. The cytosol and nucleus fraction were prepared, and the levels of p65 translocation were analyzed by western blot. (**C**) Cells were pretreated with/without RTA 408 (0.3 µM) for 4 h, and then incubated with IL-1β (0.5 ng/mL) for the indicated time intervals (15 and 30 min), ChIP assay was performed by using a specific antibody of p65. (**D**) The p65 translocation was confirmed by immunofluorescence staining. Data analysis and processing are described in the section “Statistical Analysis of Data.” * indicates *p* < 0.05; # indicates *p* < 0.01, as compared between the indicated groups. The figure represents one of three individual experiments.

To determine whether RTA 408 inhibits IL-1β-induced MMP-9 gene expression through suppressing the activation of the transcription factor NF-κB, the activation of NF-κB was determined in RBA-1 cells. As shown in Figure 6A, the IL-1β-stimulated phosphorylation of p65 was attenuated by pretreatment with RTA 408. Moreover, the nuclear translocation of p65 was detected in RBA-1 cells challenged with IL-1β. We found that IL-1β stimulated p65 translocation from cytosolic to nuclear fractions within 15–30 min, and this effect was blocked by RTA 408 pretreatment (Figure 6B). These results were further supported by the immunofluorescence images of p65 obtained using a fluorescence microscope (Figure 6D). Further, to confirm that the interaction between p65 and the MMP-9 promoter is blocked by RTA 408, a ChIP assay was performed. As shown in Figure 6C, the IL-1β-stimulated interaction between p65 and the MMP-9 promoter was indeed blocked by RTA 408. According to the above data, the inhibition of IL-1β-induced MMP-9 expression by RTA 408 was mediated by an interruption of the interaction between the MMP-9 promoter and AP-1 or NK-κB in RBA-1 cells.

### 2.6. RTA 408 Inhibits IL-1β-Induced Astroglial Cell Migration and Astrogliosis

**Figure 7:**
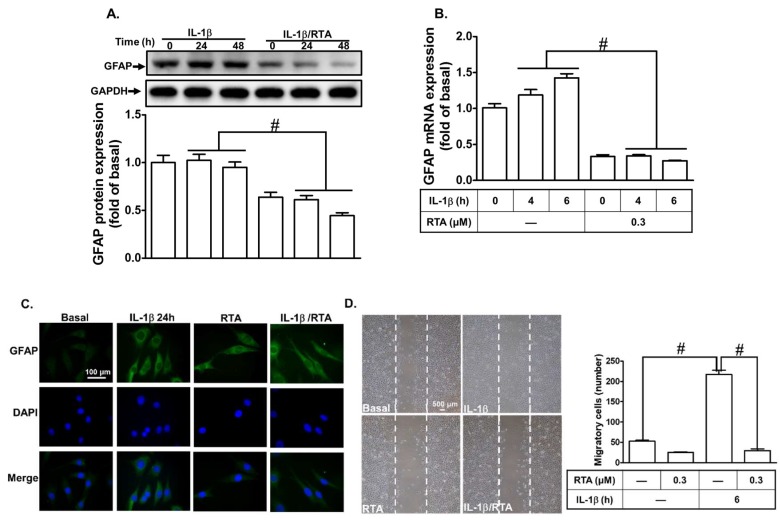
RTA 408 rescues IL-1β-triggered cell migration and astrogliosis in RBA-1 cells. (A, B) Cells were pretreated with or without RTA 408 (0.3 µM) for 4 h, and then incubated with IL-1β (0.5 ng/mL) for the indicated time intervals (24 and 48 h for protein level; 4 and 6 h for mRNA). The levels of glial fibrillary acidic protein (GFAP) protein and mRNA were assayed by (**A**) western blot using an anti-GFAP or glyceraldehyde-3-phosphate dehydrogenase (GAPDH) antibody and by (**B**) real-time PCR, respectively. (**C**) Cells were pretreated with or without RTA 408 (0.3 µM) for 4 h, and then incubated with or without IL-1β (0.5 ng/mL) for 24 h. Then, the cells were paraffin-embedded. GFAP was detected with an anti-GFAP antibody and followed with an AlexaFluor^®^488-conjugated secondary antibody (green); nuclei were stained with DAPI (blue). (**D**) Cell migration was investigated by wound healing assay. Cells were pretreated with or without RTA 408 (0.3 µM) for 4 h, and hydroxyurea (10 µM) for 1 h, and then incubated with or without IL-1β for 48 h. The images of migratory cells from the wound boundary were photographed with a digital camera under a light microscope. Data analysis and processing are described in the section “Statistical Analysis of Data.” # indicates *p* < 0.01, as compared between the indicated groups. The figure represents one of three individual experiments.

Reactive gliosis and astroglial cell migration are characteristics of neuroinflammation triggered by various inflammatory mediators [42,43]. They play a key role in the pathogenesis of brain inflammation [44]. Cytokines, including TNF-α and IL-1β, have been shown to mediate reactive astrogliosis, which is reflected by the expression of glial fibrillary acidic protein (GFAP) and cell migration, in neurodegenerative diseases [45]. Thus, we investigated whether RTA 408 exerts a neuroprotective effect by suppressing the levels of GFAP expression and cell migration induced by IL-1β. As shown in Figure 7A,B, IL-1β induced GFAP protein and mRNA expression, which was significantly attenuated by pretreatment with RTA 408. These results were further confirmed by immunofluorescence images of GFAP, and the effect was attenuated by pretreatment with RTA 408 (Figure 7C). Moreover, we investigated whether RTA 408 can block the migration of RBA-1 cells stimulated by IL-1β. As shown in Figure 7D, IL-1β increased the number of migrating cells, which was reduced by pretreatment with RTA 408. These results suggest that, in RBA-1 cells, RTA 408 attenuates IL-1β-induced cell migration and astrogliosis by suppressing MMP-9 and GFAP expression.

## 3. Discussion

**Figure 8:**
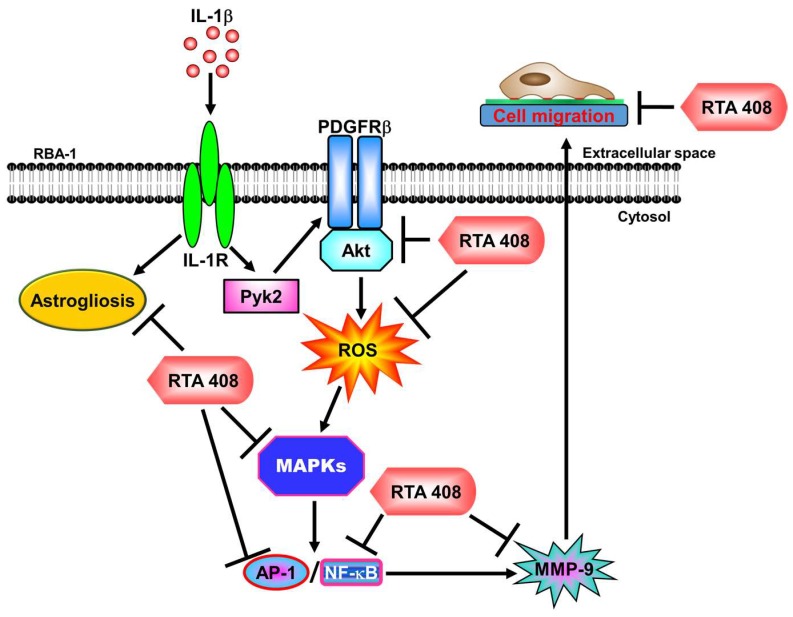
The schematic signaling pathways involved in RTA 408 inhibiting the IL-1β-induced responses in RBA-1 cells. RTA 408 attenuates Pyk2, PDGFRβ, Akt, MAPKs (p42/p44 MAPK, p38 MAPK, and JNK1/2) phosphorylation and subsequently decreases NF-κB and AP-1 activation, ultimately leading to inhibit MMP-9 expression and RBA-1 cell migration. RTA 408 also inhibits IL-1β-induced astrogliosis. “→” means “activated”; “⊥” means “inhibited”.

IL-1β has been shown to induce MMP-9 expression through ROS, protein kinases, and transcription factors in various types of cells [9,38,40]. The upregulation of MMPs triggered by cytokines plays a key role in neurodegenerative diseases. Therefore, the development of drugs targeting the expression of MMP-9 and its upstream signaling components may be beneficial for the management of inflammatory brain diseases. RTA 408 has been shown to exhibit anti-inflammatory effects partially through inhibiting NF-κB activity, increasing the levels of Nrf2-targeted gene expression, and stimulating ROS generation, although the mechanisms of the anti-inflammatory effects of RTA 408 are not fully understood. Thus, we investigated the anti-inflammatory effects of RTA 408 on IL-1β-mediated inflammatory responses in RBA-1 cells. We revealed that RTA 408 attenuates IL-1β-induced MMP-9 expression, at least in part via suppressing the phosphorylation of protein kinases, including Pyk2, PDGFR β, Akt, ROS, and MAPKs (p42/p44 MAPK, p38 MAPK, and JNK1/2), and the transcriptional activation of NF-κB and AP-1 in RBA-1 cells (Figure 8). Moreover, RTA 408 protects against IL-1β-induced neuroinflammatory responses, including cell migration and GFAP expression in astrogliosis. Although the detailed mechanisms of the in vivo and in vitro anti-inflammatory effects mediated by RTA 408 need to be explored, its anti-inflammatory and anti-oxidant effects may be useful for protecting against neurodegeneration.

MMP-9 expression has been demonstrated to be mediated by the transactivation of PDGFR and nonreceptor tyrosine kinases (Pyk2 and c-Src) induced by IL-1β, TNF-α, and lipoteichoic acid in various types of cells [46,47,48]. A previous study revealed that baicalin inhibits the PDGF-stimulated proliferation of vascular smooth muscle cells by suppressing the activation of PDGFRβ-p42/p44 MAPK signaling [49]. Our previous study also showed that Helminthostachys zeylanica extracts could inhibit bradykinin (BK)-induced MMP-9 expression via blocking Pyk2 phosphorylation in brain astrocytes [50]. Therefore, we investigated whether the inhibitory effects of RTA 408 on IL-1β-induced MMP-9 expression are mediated by suppressing the phosphorylation of Pyk2 and PDGFR in RBA-1 cells. In agreement with these reports, our results confirmed that RTA 408 inhibits IL-1β-induced MMP-9 expression by blocking Pyk2 and PDGFR phosphorylation in RBA-1 cells. Moreover, Pyk2, a nonreceptor tyrosine kinase, plays a critical role in cell migration during wound healing, suggesting that RTA 408 blocks IL-1β-induced MMP-9 expression and cell migration by attenuating the Pyk2/PDGFR cascade in RBA-1 cells.

PI3K is a heterodimeric protein consisting of a p85 regulatory subunit and a p110 catalytic subunit. Akt, a downstream component of the PI3K pathway, is important for the regulation of fundamental cellular functions, including transcription, translation, proliferation, growth, and survival. PI3K/Akt also plays a critical role in the regulation of IκB-kinase (IKK)/NF-κB activation. Thus, PI3K/Akt play key roles in the pathogenesis of various inflammatory responses. It has been noted that the expression of MMP-9 is modulated by the activation of PI3K/Akt, MAPKs, and NF-κB in brain astrocytes [9]. Curcumin can downregulate the Akt signal and cause tumor growth arrest and cell death [51]. CDDO-Me (C-28 methyl ester of 2-cyano-3,12- dioxooleana-1,9 (11)-dien-28-oic acid), a synthetic triterpenoid derived from oleanolic acid, can inhibit Akt signaling [52]. Our previous report revealed that galangin blocks thrombin-induced MMP-9 expression and cell migration via inhibiting Akt phosphorylation in SK-N-SH cells [53]. In this study, we confirmed that the inhibition of MMP-9 expression by RTA 408 is mediated by suppressing Akt activation and protects against IL-1β-mediated inflammatory responses in RBA-1 cells.

ROS act as second messengers and trigger protein kinases and transcription factors associated with various pathological and physiological functions. The generation of ROS and the induction of oxidative stress by nicotinamide adenine dinucleotide phosphate (NADPH) oxidase (NOX) and mitochondria play a key role in brain diseases [54,55,56,57]. Glycine, cannabinoids, and melatonin have been shown to block the expression of inflammatory mediators via inhibiting ROS production [54,57,58]. We also discovered that Helminthostachys zeylanica extracts might possess anti-inflammatory capability by reducing ROS-dependent MMP-9 expression in brain astrocytes challenged with BK [50]. RTA 408 has been shown to suppress NOX activity and mitochondria-ROS generation and protect against brain inflammation [29]. Therefore, the attenuation of ROS production is an effective anti-neurodegenerative strategy of in the CNS. In our study, we found that pretreatment with RTA 408 inhibits the generation of ROS by NOX that is involved in IL-1β-induced MMP-9 expression and cell migration in RBA-1 cells. Therefore, RTA 408 may be a potential anti-oxidant agent to protect against brain inflammatory diseases.

MAPKs are downstream components of ROS and relay signaling from the cell surface to the nucleus. MAPKs have been implicated in the regulation of many cellular responses, such as inflammation, proliferation, differentiation, and apoptosis. The phosphorylation of MAPKs has been shown to induce NF-κB and AP-1 activation and initiate pro-inflammatory responses [38,40]. Previous studies have also shown that IL-1β stimulates MAPKs activation, leading to the expression of MMP-9 in various types of cells [8,38]. Some herbs have been shown to exert anti-inflammatory effects on various inflammatory responses via suppressing the activation of MAPKs [59,60,61]. We have also demonstrated that galangin and Helminthostachys zeylanica extracts attenuate MAPKs-dependent MMP-9 expression in neurons and glia [50,53]. Therefore, we determined whether RTA 408 attenuates the IL-1β-stimulated phosphorylation of MAPKs in RBA-1 cells. Our results confirmed that RTA 408 reduces the IL-1β-stimulated phosphorylation of MAPKs (p42/p44 MAPK, p38 MAPK, and JNK1/2) in these cells, suggesting that the inhibitory effect of RTA 408 on IL-1β-induced MMP-9 expression and cell migration is mediated by suppressing the phosphorylation of MAPKs in RBA-1 cells.

NF-κB and AP-1 play pivotal roles in inflammatory responses, immunological reactions, and tumorigenesis. Many reports have indicated that the activation of NF-κB is involved in brain injury and inflammatory reactions. NF-κB transcription factors play a critical role in MMP-9 induction and are regarded as therapeutic targets for the treatment of various inflammatory diseases. IL-1β has been reported to evoke the expression of MMP-9 via the activation of NF-κB [9,40]. RTA 408 can inhibit NF-κB signaling and attenuate inflammatory responses in RAW 264.7 macrophage cells [22]. Moreover, the herb extracts exert their anti-inflammatory effect by blocking the activation of NF-κB [59,62]. Our data also demonstrated that RTA 408 inhibits IL-1β-stimulated p65 phosphorylation and translocation from cytoplasmic to nuclear fractions. Moreover, RTA 408 also attenuated the association of NF-κB with binding sites on the MMP-9 promoter. These results confirmed that RTA 408 inhibits IL-1β-induced MMP-9 expression and cell migration by blocking NF-κB activation in RBA-1 cells.

AP-1 regulates the expression of inflammatory genes in response to different stimuli, including cytokines, growth factors, stress signals, and bacterial and viral infections. AP-1 is a crucial component for MMP-9 induction and a candidate for the treatment of inflammatory brain diseases. Our previous study revealed that IL-1β-induced MMP-9 expression is mediated by AP-1 activation in RBA-1 cells [9,40]. Moreover, several herb extracts have anti-inflammatory effects by blocking the activity of AP-1 [63,64]. Our recent studies have also demonstrated that galangin and Helminthostachys zeylanica extracts attenuate proinflammatory mediator-induced MMP-9 expression via inhibiting the activity of AP-1 [50,53]. Therefore, we determined whether RTA 408 attenuates the IL-1β-stimulated activity of AP-1 in RBA-1 cells. In the present study, RTA 408 suppressed IL-1β-stimulated AP-1 activation and c-Jun mRNA expression and thereby attenuated MMP-9 expression and cell migration in RBA-1 cells.

## 4. Materials and Methods

### 4.1. Materials

Dulbecco’s modified Eagle’s medium (DMEM)/Ham’s nutrient mixture F-12 (F-12) and fetal bovine serum (FBS) were purchased from Invitrogen (Carlsbad, CA, USA). Hybond C membranes and an enhanced chemiluminescence (ECL) detection system were obtained from GE Healthcare Biosciences (Buckinghamshire, UK). Anti-phospho-Akt (Ser^473^, #9271), anti-phospho-Pyk2 (Tyr^402^, #3291), anti-phospho-PDGFRβ (Tyr^751^, #3161), anti-phospho-p38 MAPK (Thr^180^/Tyr^182^, #9211), anti-phospho-c-Jun amino-terminal kinase (JNK)1/2 (Thr^183^/Tyr^185^, #4668), anti-phospho-extracellular signal-regulated kinase 1/2 (Erk1/2; Thr^202^/Tyr^204^, #9101), anti-phospho-p65 (Ser^536^, #3031), anti-phospho-c-Jun (Ser^63^, #2361), and anti-p47^phox^ (#4301) antibodies were obtained from Cell Signaling (Danvers, MA, USA). Anti-Pyk2 (ab32448) and anti-Nox2 (ab129068) antibodies were purchased from Abcam (Cambridge, UK). Anti-glyceraldehyde-3-phosphate dehydrogenase (GAPDH) (#MCA-ID4) was purchased from Encor (Gainesville, FL, USA). Anti-lamin A (sc-20680), anti-p47^phox^ (sc-14015), anti-Gαs (sc-823), anti-PDGFRβ (sc-374573), anti-p38 (sc-535), anti-Akt (sc-8312), anti-ERK1 (sc-271270), anti-ERK2 (sc-1647), anti-JNK (sc-7345), anti-p65 (sc-398442), anti-c-Jun (sc-44), and anti-GFAP (sc-33673) antibodies were purchased from Santa Cruz (Santa Cruz, CA, USA). All primary antibodies were diluted at 1:1000 in PBS with 1% bovine serum albumin (BSA). N-(2-cyano-3,12-dioxo-28-noroleana-1,9(11)-dien-17-yl)-2-2-difluoropropanamide (RTA 408) was purchased from Cayman Chemical (Ann Arbor, MI, USA). Bicinchoninic acid (BCA) protein assay reagent was purchased from Pierce (Rockford, IL, USA). Sodium dodecyl sulfate (SDS)-PAGE reagents were purchased from MDBio, Inc. (Taipei, Taiwan). Dimethyl sulfoxide (DMSO), IL-1β, enzymes, TRIzol, a sodium 3′-[1-[(phenylamino)-carbony]-3,4-tetrazolium]-bis(4-methoxy-6-nitro)benzene-sulfonic acid hydrate (XTT) assay kit, and other chemicals were obtained from Sigma (St. Louis, MO, USA). CellROX Deep Red Reagent and 2′,7′-dichlorodihydrofluorescein diacetate (DCFH-DA) were obtained from ThermoFisher Scientific (Waltham, MA, USA).

### 4.2. Cell Culture and Treatment

RBA-1 cell line originating from the neonatal rat cerebrum was kindly provided by Professor T.C. Jou at the Institute of Neroscience, National Yang Ming University (Taipei, Taiwan) [65] and used throughout this study. The purity of the cultured astrocytes was assessed using an astrocyte-specific marker, anti-glial fibrillary acidic protein (GFAP) antibody, which showed that over 95% of the astrocytes were GFAP-positive. Experiments were performed with cells from passages 4 to 35. The cytotoxicity of RTA 408 or IL-1β alone at the time of incubation was checked using an XTT assay kit, and these treatments were found to have no significant effect on cell viability. The cells were plated in 12-well culture plates, made quiescent at confluence by incubation in serum-free DMEM/F-12 for 24 h, and then incubated with IL-1β at 37 °C for the indicated time intervals. When RTA 408 or other inhibitors were used, the cells were pretreated with RTA 408 or other inhibitors for the indicated time intervals before exposure to IL-1β.

### 4.3. Protein Preparation and Western Blotting

The procedure was conducted as previously described [66]. Briefly, after cells were washed with ice-cold PBS, whole-cell extracts were collected, and buffer containing 0.1 M Tris-HCl (pH 6.8), 1% SDS, 5% glycerol, 2.5% β-mercaptoethanol, and 0.02% bromophenol blue was used to collect whole-cells extracts. Western blotting was performed by using SDS-PAGE. Proteins were transferred by electrophoresis onto Hybond-C membranes which were incubated with a specific antibody diluted at 1:1000 with Tween-Tris buffered saline and incubated with an anti-GAPDH antibody as an internal control. The membranes were washed four times with Tween-Tris buffered saline in 30 min. The membranes were immersed in a horseradish peroxidase-conjugated secondary antibody (1:1500 dilution) for 1 h. The membranes were washed four times with Tween-Tris buffered saline in 30 min. The immunoreactive bands were detected by ECL and captured using a UVP BioSpectrum 500 Imaging System (Upland, CA, USA). UN-SCAN-IT gel software version 6.1 (Silk Scientific Inc., Orem, UT, USA) was used to quantify the image densitometry analyses.

### 4.4. MMP Gelatin Zymography

The procedure was conducted as previously described [42]. Briefly, growth-arrested cells were incubated with IL-1β for the indicated time intervals. The culture media were collected and analyzed by gelatin zymography. The horizontal white bands on a blue background represented the gelatinolytic activity of MMP-9. Because cleaved MMPs were not reliably visible, we only measured pro-form zymogens.

### 4.5. Total RNA Extraction and Real-Time PCR Analysis

The procedure was conducted as previously described [38]. Briefly, total RNA was extracted from RBA-1 cells. The template for PCR amplification used the cDNA obtained from 0.5 μg of total RNA. Based on GenBank entries for rat MMP-9 and GAPDH, oligonucleotide primers were designed. The designed primers were as follows: MMP-9, 5′-AGTTTGGTGTCGCGGAGCAC-3′ (sense), 5′-TACATGAGCGCTTCCGGCAC-3′ (antisense); GAPDH, 5′-AACTTTGGCATCGTGGAAGG-3′ (sense), 5′-GTGGATGCAGGGATGATGTTC-3′ (antisense). The TaqMan gene expression assay system with primers and probe mixes for MMP-9 and endogenous GAPDH control genes was used for real-time PCR. PCR was performed using the 7500 Real-Time PCR System (Applied Biosystems, Foster City, CA, USA). The ΔΔCt method was used to determine the relative gene expression, where Ct represented the threshold cycle. All experiments were performed in triplicate.

### 4.6. Rat MMP-9 Promoter Construction, Transfection, and Luciferase Reporter Gene Assays

The method was conducted as previously described [67,68]. Briefly, the upstream region (−1280 to +19) of the rat MMP-9 promoter was cloned. The QIAGEN (Hilden, Germany) plasmid DNA preparation kits were used to prepare the plasmid. The construct was transfected by using Lipofectamine reagent. Transfection with an enhanced green fluorescent protein (GFP) was used to determine the transfection efficiency (~60%). After incubation with IL-1β, the cells were collected, sonicated, and centrifugated. The aliquots of the supernatants were assayed for promoter activity using a luciferase assay system (Promega, Madison, WI, USA). Firefly luciferase activity was standardized to β-galactosidase activity.

### 4.7. Isolation of Cell Fractions

After incubation, the cells were harvested, sonicated for 5 s at output 1.5 with a sonicator (Misonix, Inc., Farmingdale, NY, USA), and centrifuged at 8000 rpm for 15 min at 4 °C to yield the pellet (nuclear fraction) and the supernatant (cytosolic fraction).

### 4.8. Measurement of Intracellular ROS Accumulation

Intracellular H_2_O_2_ levels were determined by measuring the fluorescence of DCFH-DA. For the purpose of these experiments, the cells were plated in six-well culture plates with coverslips. The cells were treated with IL-1β with/without pretreatment with RTA 408 for the indicated time intervals (5 and 10 min). The cells were washed with warm PBS and incubated in PBS containing 10 μM DCFH-DA at 37 °C for 30 min. Subsequently, the PBS containing DCFH-DA was removed and replaced with fresh medium. The cells were washed twice with PBS and then observed by using a fluorescence microscope (Zeiss, Axiovert 200M). In addition, CellROX Deep Red Reagent was added to the cells at a final concentration of 5 μM and then incubated for 30 min at 37 °C. Subsequently, the medium was removed, and the cells were washed three times with PBS. The resulting fluorescence was measured using a fluorescence microscope.

### 4.9. Immunofluorescence Staining

Growth-arrested cells were treated with IL-1β (0.5 ng/mL) for the indicated time intervals. After washing twice with ice-cold PBS, cells were fixed, permeabilized, stained using anti-GFAP and anti-p65 antibodies (1:200 dilutions), and finally mounting as previously described [35]. Then, 4′,6-diamidino-2-phenylindole (DAPI) was used to stain the DNA. The images were observed with a fluorescence microscope (Zeiss, Axiovert 200 M).

### 4.10. Cell Migration Assay

RBA-1 cells were cultured to confluence in six-well culture plates and starved in serum-free DMEM/F-12 medium for 24 h. The monolayer cells were manually scratched with a blue pipette tip to create extended and definite scratches with a bright and clear field (~2 mm) in the center of the dishes. The detached cells were removed by washing the cells once with PBS. Serum-free DMEM/F-12 medium with or without IL-1β (0.5 ng/mL) and containing the DNA synthesis inhibitor hydroxyurea (10 μM) was added to each dish during the period of observation as indicated after pretreatment with or without RTA 408 for 1 h. Migrating cells from the scratch boundary were observed under a light microscope and imaged with a digital camera (Olympus, Japan). Data analysis and processing were conducted as in our previous study [66]. The data demonstrated were summarized from three individual experiments.

### 4.11. Chromatin Immunoprecipitation (ChIP) Assay

Chromatin immunoprecipitation analysis was conducted as previously described [68] to detect the in vivo association of nuclear proteins with the rat MMP-9 promoter. Briefly, RBA-1 cells were cross-linked with 1% formaldehyde for 10 min at 37 °C and washed three times with ice-cold PBS containing 1 mM phenylmethylsulfonyl fluoride (PMSF) and 1% aprotinin. The cell lysates were prepared using SDS-lysis buffer (1% SDS; 5 mM EDTA; 1 mM PMSF; and 50 mM Tris-HCl) and were sonicated at 4 °C until the DNA size was 200–300 base pairs. After the soluble chromatin was precleared by incubation with sheared salmon sperm DNA-protein agarose A, one portion of the sample was used as the DNA input control, and the other portion of supernatant was immunoprecipitated without (control) or with anti-p65 or anti-c-Jun antibodies and protein A beads. Following washes and elution, the precipitates were heated overnight at 65 °C to reverse the cross-linking of the DNA and protein. The DNA fragments were purified by phenol-chloroform extraction and ethanol precipitation. The purified DNA was subjected to PCR amplification using the primers specific for the region (−606 ~ 327, accession NO: AF148065) containing the distal AP-1 binding site (−503 to −497) and p65 binding site (−560 to −550) present in the MMP-9 promoter region (sense primer: 5′-AGAGCCTGCTCCCAGAGGGC-3′; antisense primer: 5′-GCCAAGTCAGGCAGGACCCC-3′). The PCR fragments were analyzed on gels containing 3% agarose in 1× TAE and ethidium bromide, and the size of the fragments (279 bp) was compared to a molecular weight marker.

### 4.12. Statistical Analysis of Data

All data are expressed as the mean or mean ± SEM of three individual experiments performed in duplicate or triplicate. For the western blot data, the significance of the differences between the two groups was determined by a paired two-tailed Student’s *t*-test. All other statistical analyses compared multiple groups. GraphPad Prism version 5.01 (GraphPad, San Diego, CA, USA) was used to analyze the data by an unpaired *t*-test or one-way analysis of variance [69], followed by Tukey’s post hoc test. * *p* < 0.05; ^#^ *p* < 0.01, compared with the cells stimulated with IL-1β only or compared with the indicated groups.

## 5. Conclusions

In summary, reactive astrogliosis may be a potential target for therapeutic interventions for neurodegenerative disorders. Several studies have shown the relationship between MMP-9 expression and astrogliosis [70,71,72]. Here, our results suggest that RTA 408 suppresses IL-1β-induced astrogliosis and cell migration in vitro. Further, it is worth investigating the mechanisms underlying the effects of RTA 408 on astrogliosis in animals. These results provide new insights into the mechanisms by which RTA 408 attenuates MMP-9 expression induced by IL-1β. Moreover, an increased understanding of the anti-inflammatory effects of RTA 408 creates opportunities for the development of potential anti-inflammatory therapeutic strategies for neurodegeneration and neuroinflammation.

## Abbreviations

AD	Alzheimer’s disease
AP-1	activator protein 1
BBB	blood-brain barrier
BCA	bicinchoninic acid
BK	bradykinin
BSA	bovine serum albumin
CDDO-Me	C-28 methyl ester of 2-cyano-3,12- dioxooleana-1,9 (11)-dien-28-oic acid
ChIP	chromatin immunoprecipitation
CNS	central nervous system
DAPI	4′,6-diamidino-2-phenylindole
DCFH-DA	2′,7′-dichlorodihydrofluorescein diacetate
DMEM	Dulbecco’s modified Eagle’s medium
DMSO	dimethyl sulfoxide
ECL	enhanced chemiluminescence
EDTA	ethylenediaminetetraacetic acid
ERK1/2	extracellular signal-regulated kinase 1/2
FBS	fetal bovine serum
GAPDH	glyceraldehyde-3-phosphate dehydrogenase
GFAP	glial fibrillary acidic protein
GFP	green fluorescent protein
IKK	IκB-kinase
IL-1β	interleukin-1β
JNK	c-Jun amino-terminal kinase
MAPK	mitogen-activated protein kinase
MMP	matrix metalloproteinase
NF-κB	nuclear factor-κB
NOX	NADPH oxidase
PD	Parkinson’s disease
PDGFR	platelet-derived growth factor receptor
PI3K	phosphoinositide 3-kinase
PMSF	phenylmethylsulfonyl fluoride
Pyk2	proline-rich tyrosine kinase
RBA-1	rat brain astrocytes
ROS	reactive oxygen species
RTA 408	N-(2-cyano-3,12-dioxo-28-noroleana-1,9(11)-dien-17-yl)-2-2-difluoropropanamide
SEM	standard error of the mean
SDS	Sodium dodecyl sulfate
TNF	tumor necrosis factor
XTT	sodium 3′-[1-[(phenylamino)-carbony]-3,4-tetrazolium]-bis(4-methoxy-6-nitro)benzene-sulfonic acid hydrate
